# Molecular typing tools for identifying and characterizing lactic acid bacteria: a review

**DOI:** 10.1007/s10068-020-00802-x

**Published:** 2020-08-16

**Authors:** Anshul Sharma, Sulhee Lee, Young-Seo Park

**Affiliations:** 1 Department of Food and Nutrition, Gachon University, Seongnam, 13120 Republic of Korea; 2 Faculty of Applied Sciences and Biotechnology, Shoolini University of Biotechnology and Management Sciences, Bajhol, Solan, Himachal Pradesh 173229 India; 3 Research Group of Healthcare, Korea Food Research Institute, Wanju, 55365 Republic of Korea; 4 Department of Food Science and Biotechnology, Gachon University, Seongnam, 13120 Republic of Korea

**Keywords:** Lactic acid bacteria, Probiotics, DNA, RNA, Fingerprinting, Typing

## Abstract

Identification and classification of beneficial microbes is of the highest significance in food science and related industries. Conventional phenotypic approaches pose many challenges, and they may misidentify a target, limiting their use. Genotyping tools show comparatively better prospects, and they are widely used for distinguishing microorganisms. The techniques already employed in genotyping of lactic acid bacteria (LAB) are slightly different from one another, and each tool has its own advantages and disadvantages. This review paper compiles the comprehensive details of several fingerprinting tools that have been used for identifying and characterizing LAB at the species, sub-species, and strain levels. Notably, most of these approaches are based on restriction digestion, amplification using polymerase chain reaction, and sequencing. Nowadays, DNA sequencing technologies have made considerable progress in terms of cost, throughput, and methodology. A research journey to develop improved versions of generally applicable and economically viable tools for fingerprinting analysis is ongoing globally.

## Overview

There is a growing interest in the identification of industrially relevant and beneficial microbial strains owing to their heterogeneity and ubiquitous nature. Simple morphological characterization of these microorganisms is ineffective in documenting a complete diversity profile (Tabssum et al., 2018). Over time, many typing tools, phenotypic or genotypic, have been documented; however, an effective tool is preferred to have high typeability and discriminatory power for the microorganisms that are under study (Ben Amor et al., 2007). Among the beneficial microbes, probiotics are live microorganisms, which confer health benefits on the host when administered in adequate amounts (FAO/WHO 2002). Probiotics have been found to have beneficial effects on human and animal health (Stefanis et al., 2016), and generally are some lactic acid bacteria (LAB), including *Lactobacillus* (*Lb.*) *acidophilus*, *Lb. casei* (recently reclassified as *Lacticaseibacillus casei*, Zheng et al., 2020), *Lb. fermentum* (recently reclassified as *Limosilactobacillus fermentum*, Zheng et al., 2020), *Lb. helveticus*, *Lb. paracasei* (recently reclassified as *Lacticaseibacillus paracasei*, Zheng et al., 2020), *Lb. plantarum* (recently reclassified as *Lactiplantibacillus plantarum*, Zheng et al., 2020), *Lb. reuteri* (recently reclassified as *Limosilactobacillus reuteri*, Zheng et al., 2020), *Lb. rhamnosus* (recently reclassified as *Lacticaseibacillus rhamnosus*, Zheng et al., 2020), *Lb. salivarius* (recently reclassified as *Ligilactobacillus salivarius*, Zheng et al., 2020), *Lb. lactis* subsp. *cremoris*, *Lb. lactis* subsp. *lactis*, *Streptococcus (St.) thermophilus,* and some bifidobacteria including *Bifidobacterium (Bif.) animalis, Bif. bifidum*, *Bif. breve*, and *Bif. longum*. Among the yeasts, *Saccharomyces (S.) carlsbergensis*, *S. cerevisiae, S. lactis,* and *S. rouxii* are also probiotic strains. Interestingly, some LAB strains are generally recognized as safe (GRAS) microorganisms. LAB are rod- or cocci-shaped Gram-positive bacteria with low G+C content and common morphological, physiological, and metabolic characteristics (Wu et al., 2017). LAB are common inhabitants of the human gastrointestinal tract, and are omnipresent in fermented and unfermented foods. The wide range and number of applications of LAB make it necessary to associate genomic proof with their important features to exploit their metabolic applications (Stefanovic et al., 2017; Wu et al., 2017) and identify novel microorganisms at all taxonomic levels rapidly and precisely (Jarocki et al., 2016). Furthermore, there is a need for new starter species from the wild LAB pool for generating diverse food and pharmaceutical products that target human health (Sharma et al., 2020).

The identification and classification of LAB populations, based on traditional phenotypic, biochemical, and physiological tests, is well known. Classification of some new types of strains based merely on phenotypic characteristics has caused obscurities, which have eventually become fixed by means of molecular tools (Van Hoorde et al., 2008). Furthermore, it is extremely difficult to identify a bacterial strain using these approaches due to the several complicated procedures, different nutritional and growth needs of LAB, and lower discriminatory power (Østlie et al., 2004; Singh et al., 2009). Therefore, such inadequacies of the phenotypic tools led to the emergence of genotypic methods to classify LAB. The molecular typing tools can be broadly categorized as polymerase chain reaction (PCR) amplification-, DNA-, and sequencing-based tools (Ranjbar et al., 2014). Furthermore, a critical step for the molecular documentation of LAB is choosing the appropriate genetic marker or gene that can be used for PCR amplification to discriminate the LAB species (Pogačić et al., 2010). The major advantage of the DNA-based methodologies is that they accurately identify the strains.

DNA sequencing skills have made significant strides in the past decade in terms of price per reaction and user handiness. Nonetheless, phenotypic tools are important for the initial classification of formerly unidentified LAB species. Overall, a polyphasic approach that integrates several lines of evidence should be used to acquire a comprehensive account of a new LAB species.

This review is aimed to summarize current knowledge on the molecular tools that are used to identify and characterize several bacterial species, including LAB and probiotic strains. Exemplary studies, based on these tools for typing of LAB or probiotic strains, have also been included. Furthermore, this paper briefly includes the advantages and disadvantages of each fingerprinting method.

## Types of molecular tools

Since the mid-1980s, the plethora of various molecular techniques has been widely applied to characterize probiotic or LAB species. Each technology has its own strengths, usefulness, and drawbacks. Notably, there is no single method that can provide all the information on the inter- and intra-species differentiation. Therefore, the current strategy is to follow a multiphasic approach to identify and characterize LAB strains correctly. Contrary to phenotypic approaches, molecular tools are faster and far more dependable and reproducible, and can even differentiate among closely related species, which are otherwise phenotypically indistinguishable.

## Ribotyping

**Fig. 1:**
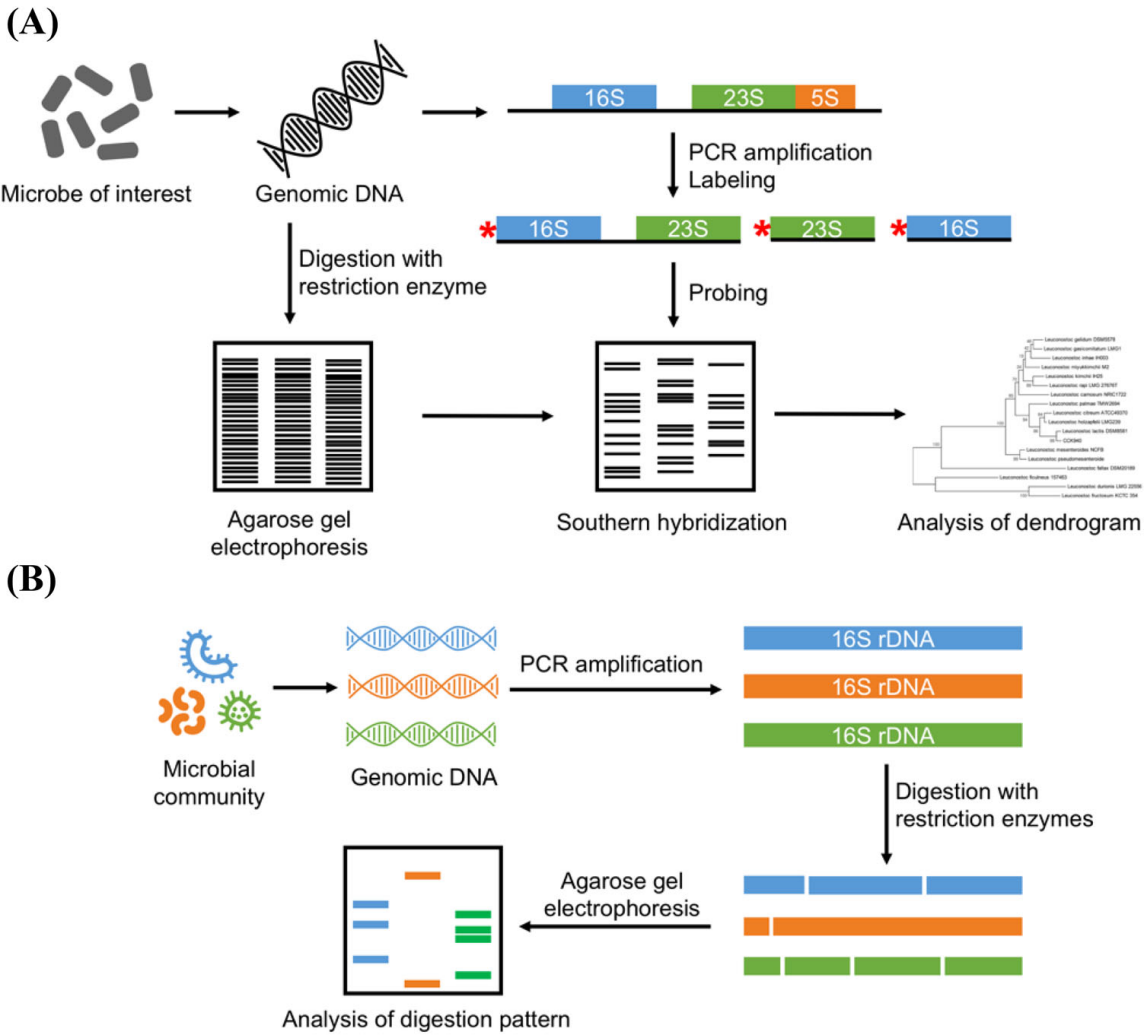
Schematic diagram for Ribotyping (**A**) and amplified ribosomal DNA restriction analysis

In this approach, ribosomal genes present within the bacterial genome are recognized using nucleic acid probes. This combined detection is based on the digestion of genomic DNA of the microbe of interest with restriction endonuclease and Southern hybridization using rDNA cistrons (16S, 23S, and 5S rRNA genes) as the labeled probes (Fig. 1(A)). The ribotyping probes range from partial sequences of the rDNA genes or their spacer regions to the full rDNA operon (Stefanis et al., 2016). Particularly, the intergenic space region between the 16S rDNA and 23S rDNA shows high polymorphism. Higher discriminatory power is obtained in those situations in which multiple ribopatterns are used for each ribotype, especially when restriction enzymes with particular recognition sequences are used. Nevertheless, ribotyping is more suitable for distinguishing bacteria at the species and subspecies levels than at the strain level (Ben Amor et al., 2007).

The ribotyping tool was used to classify strains of *Lactobacillus* sp. obtained from commercial products and human fecal samples (Giraffa et al., 2000; Zhong et al., 1998). Ribotyping has been successfully applied for the discrimination of the *Lb. casei*, *Lb. acidophilus*, *Lb. reuteri*, *Lb. sakei* (recently reclassified as *Latilactobacillus sakei*, Zheng et al., 2020), *Lb. delbrueckii*, *Lb. plantarum*, *Lb. helveticus*, *Lb. rhamnosus*, *Lb. crispatus*, *Lb. fermentum*, and *Lb. gasseri* species at the species and strain levels, as reviewed previously (Singh et al., 2009). A previous study documented the use of a riboprinter (an automated tool) for characterizing and differentiating 91 strains of the *Lb. acidophilus* and *Lb. casei* groups at the species level. The riboprinter tool was found to be fast, precise, and reproducible (Ryu et al., 2001).

The advantages of ribotyping include general applicability, reproducibility, and high discriminatory power. However, it is expensive, labor-intensive, and time-consuming. Using an automated riboprinter can be easier and faster, and it identifies microbial species in a shorter time span (8 h); hence, it significantly reduces the testing time.

## Amplified ribosomal DNA restriction analysis (ARDRA)

ARDRA, a type of restriction fragment length polymorphism (RFLP), is a simple method, and technically, it is a variation of ribotyping (Stefanis et al., 2016). This technique works on the principle of digesting amplified ribosomal DNA with selected restriction endonucleases, which are capable of cleaving DNA at specific sequences, generating fragments of different lengths, and performing size-based separation by agarose gel electrophoresis (Fig. 1(B)). In some cases, the fragments may be transferred to nitrocellulose or nylon membranes, and after that, specific probing and finally detection are performed. Deletions or insertions, mutations in restriction endonuclease sites, and the acquisition or removal of restriction endonuclease recognition sites may result in variations in the bacterial strains.

Recently, this technique was utilized to identify human gastrointestinal tract-originated 14 reference *Lactobacillus* species (Öztürk and Meterelliyöz 2015). Likewise, the *Lb. acidophilus* group was identified in the crops of birds and typed (Hagen et al., 2003). Stenico et al. identified 13 *Bifidobacterium* species by RFLP analysis of the partial gene sequence of the heat shock protein (*hsp*60) at the species and subspecies levels (Stenico et al., 2014).

This technique is fast, easy, and cost-effective. The complexity of the banding profiles is the major limitation of this method. In addition, owing to this, multiple restriction endonucleases are required either separately or in combination to obtain the desired resolution. Besides, the technique displays low discriminatory power due to the comparatively conserved nature of the 16S rRNA genes. Additionally, the technique is less sensitive with respect to gel staining, and thus, it is utilized in populations with few bacterial species.

## Random amplified polymorphic DNA (RAPD)

**Fig. 2:**
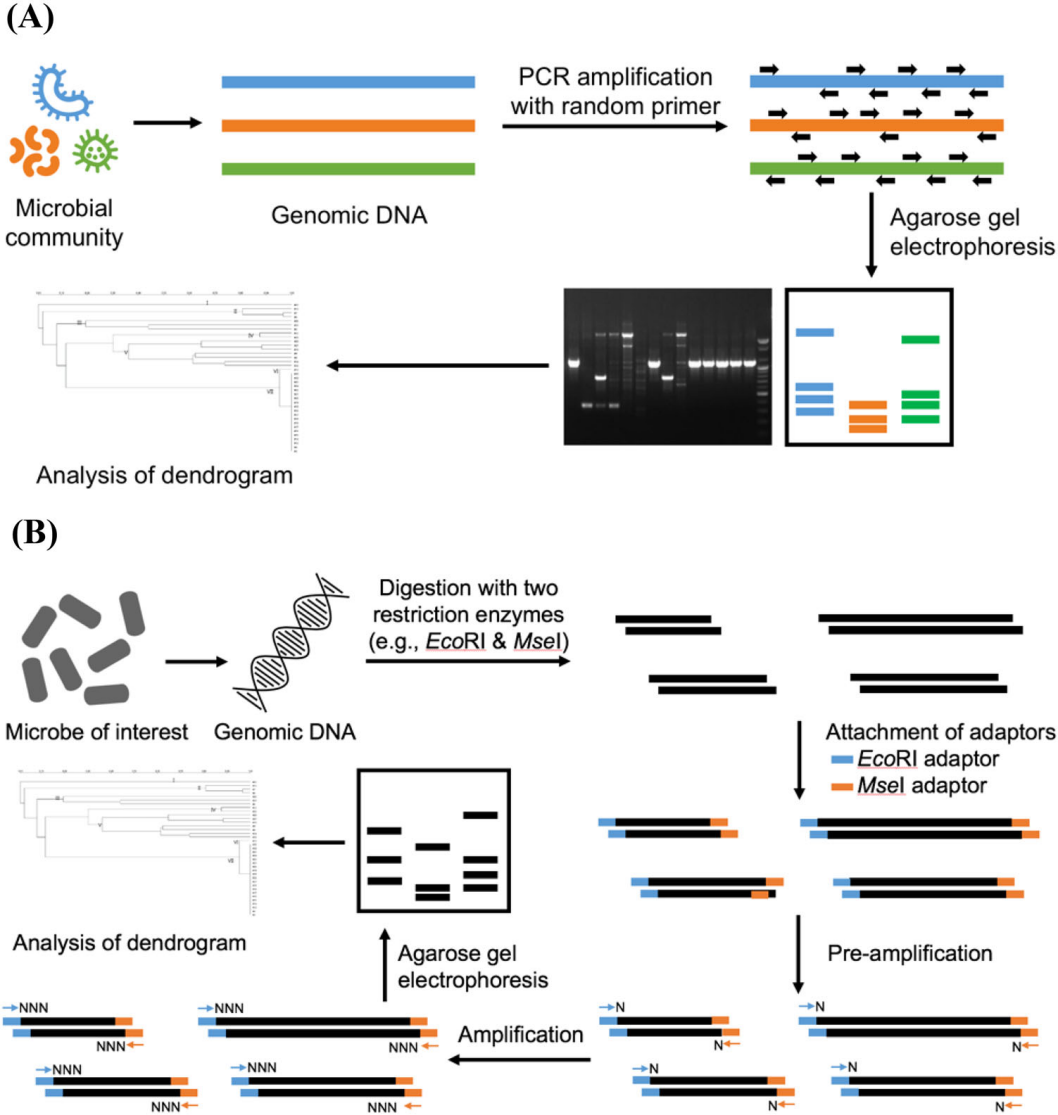
Schematic diagram for random amplified polymorphic DNA (**A**) and amplified fragment length polymorphism (**B**)

RAPD is a typing tool, which uses a single arbitrary primer of 20–25 base pairs (bps). This primer randomly hybridizes to different locations of chromosomal DNA sequences that show homology nearest to the that of the bacterial genome to detect polymorphisms (Williams et al., 1990). Variants of RAPD, based on the length of the primer, annealing temperature, and length of the protocol, include DNA amplification fingerprinting and arbitrarily primed PCR (Ranjbar et al., 2014). Agarose gel electrophoresis separates the amplification products to generate a bacterial fingerprint that is used to identify and characterize bacterial strains (Fig. 2(A)). When sequence information of the newly isolated strains is not accessible, RAPD analysis is first applied for the development of strain-specific primers (Plessas et al., 2017).

Weiss et al. used and identified RAPD patterns of the strains of *Lb. reuteri*, a potential probiotic bacterium (Weiss et al., 2005). Similarly, the RAPD technique has been used for the characterization of different strains of the *Lactobacillus*, including *Lb. plantarum* (Galanis et al., 2015), *Lb. brevis* (recently reclassified as *Levilactobacillus brevis*, Zheng et al., 2020) (Sharma et al., 2016), *Lb. acidophilus,* and *Lb. fermentum* (Kakelar et al., 2019), and *Bacillus* species (Mohkam et al., 2016). Our research group recently found that RAPD could be used as a valuable tool for identifying *Leuconostoc (Leu.) mesenteroides*, *Lb. brevis*, and *Lb. plantarum* from deliberately inoculated yogurt and a commercial probiotic powder (Sharma et al., 2016; Sharma et al., 2020). Additionally, *Lb. paracasei* K5 was identified from a Feta-type cheese using a multiplex PCR technique based on the RAPD analysis (Plessas et al., 2017).

Another study aimed to identify the enzymatic activities responsible for the degradation of biogenic amines in LAB. Through RAPD and 16S rRNA gene partial sequencing, different LAB strains, such as the *Leuconostoc* (*Leu. mesenteroides* and *Leu. lactis*), *Lactobacillus* (*Lb. casei*, *Lb. paracasei*, *Lb. rhamnosus*, *Lb. parabuchneri* (recently reclassified as *Lentilactobacillus parabuchneri*, Zheng et al., 2020), *Lb. paraplantarum* (recently reclassified as *Lactiplantibacillus paraplantarum,* Zheng et al., 2020), and *Lb. fermentum*), and *Streptococcus* species (*St. thermophilus* and *St. gallolyticus*), *Lactococcus* (*Lac.*) *lactis*, *Pediococcus* (*P.*) *pentosaceus*, *Enterococcus lactis*, and *Weissella paramesenteroides*, were identified (Guarcello et al., 2016). Our research group also identified *Leu. mesenteroides*, *Lb. brevis*, and *Leu. citreum* in different foods and food sources in Korea using RAPD markers (Kaur et al., 2017a; Sharma et al., 2016). The RAPD tool has been used for the characterization of *Bifidobacterium* strains, including *Bif. bifidum*, *Bif. infantis*, *Bif. adolescentis*, *Bif. longum, Bif. animalis*, and *Bif. breve* (Vincent et al., 1998).

The advantages of RAPD include good discriminatory power, general applicability, and fast, inexpensive, and easy performance (Ngoi et al., 2015). The discriminatory power can be enhanced by increasing the number of primers. Besides, this technique requires no prior knowledge of the target DNA sequence and a limited amount of bacterial DNA for amplification (Kaur et al., 2017a; Ranjbar et al., 2014). Poor reproducibility is the major drawback of this technique. Reproducibility depends on many factors, such as the quantity and quality of DNA, PCR buffer, primer concentration, and annealing temperature. It has been suggested that accurate optimization of the PCR protocol could overcome this problem (Manan et al., 2009). Besides, the use of triplet arbitrary primed-PCR at three different temperatures (38, 40, and 42 °C) has been advocated. Furthermore, primer hybridization at non-specific locations may give rise to false-positive results.

## Amplified fragment length polymorphism (AFLP)

AFLP was originally used for the characterization of plant genomes, but it has become more common in the field of microbial typing over time. There are two AFLP versions, one with two separate restriction endonucleases and PCR amplification primers and the other with a single primer and restriction endonuclease.

AFLP is a combination of RFLP and PCR. The target DNA is digested with restriction endonucleases, as in RFLP, and ligated with primers, known as adapters, for PCR amplification (Fig. 2(B)). The mixture is subjected to selective amplification using a limited set of primers. For the digestion of genomic DNA, AFLP uses two restriction enzymes, a frequent cutter (e.g. *Mse*I or *Taq*I) and rare cutter (e.g. *EcoR*I or *Pst*I) (Vos et al., 1995). For selective amplification to reduce the number of amplicons after digestion with restriction endonuclease, the 3’-ends of the PCR primers are modified by adding specific nucleotides (one to three). The pre-amplification (first) PCR is accomplished with first combinations that contain a single bp extension, while the selective (final) PCR amplification is achieved using primers with up to three bp extensions. These modified primers anneal to only DNA target fragments that have complementary sequences to the adaptors and modified nucleotides, allowing specific amplification. The amplified fragments undergo electrophoresis either on an agarose gel or with high resolution denaturing polyacrylamide gel with autoradiography (Vos et al., 1995). The fluorescent-labeled PCR primers have emerged as alternatives to radioactive material when an automated DNA sequencer is used. This approach offers high discriminatory power, resolution, and throughput (Giammanco et al., 2007; Ross and Heuzenroeder 2005).

The advantages of AFLP include higher reproducibility and sensibility and no requirement for prior knowledge of the sequence (Singh et al., 2009). The AFLP data are analyzed based on the presence or absence of polymorphism. This allows for a rapid scan of the entire genome for polymorphisms. The limitations of this technique include poor target DNA quality, complicated procedure with a large number of steps, expensive process, and the prerequisite of an automated DNA sequencer.

The AFLP technique has been effectively utilized for genotyping and intra-species documentation of LAB obtained from many fermented food products and the human gastrointestinal tract (Ben Amor et al., 2007). In a previous study, AFLP was used for differentiation of *Lb. plantarum, Lb. pentosus* (recently reclassified as *Lactiplantibacillus pentosus*, Zheng et al., 2020), and *Lb. pseudoplantarum* at the species level (Giraffa and Neviani 2000). Bove et al. compared *Lb. rhamnosus* isolated from cheese grown in rich medium (MRS) and cheese-like medium (CB) using complementary DNA (cDNA) AFLP. The study established that gene profiles of *Lb. rhamnosus* showed more diversity in CB than in MRS. The diverse gene expression levels in CB were plausibly due to the activation of different metabolic pathways to produce a high amount of energy (Bove et al., 2011). Another study showed that AFLP could be used as a tool to establish a correlation between the carbohydrate utilization capacities and niche/genotype adaptation of the *Lb. rhamnosus* species isolated from humans and food (Ceapa et al., 2015). Furthermore, a previous study differentiated many *Lactobacillus* strains at the intra-species level using fluorescent AFLP (fAFLP) (Vancanneyt et al., 2006). Another study designed oligonucleotide primers using a fAFLP-derived gene fragment (125 bps) that encoded the aldo/keto reductase enzyme for the species-specific PCR assay of *Lb. brevis* (Fusco et al., 2016).

## Pulse field gel electrophoresis (PFGE)

PFGE is a type of gel electrophoresis that separates large DNA molecules under pulsed-field conditions. PFGE is very similar to the typical gel electrophoresis technique. Agarose gel electrophoresis is incapable of effectively separating large DNA molecules (Chen and Gu 2018). Using PFGE, DNA fragments up to 800 kb in size can be resolved (Ranjbar et al., 2014).

**Fig. 3:**
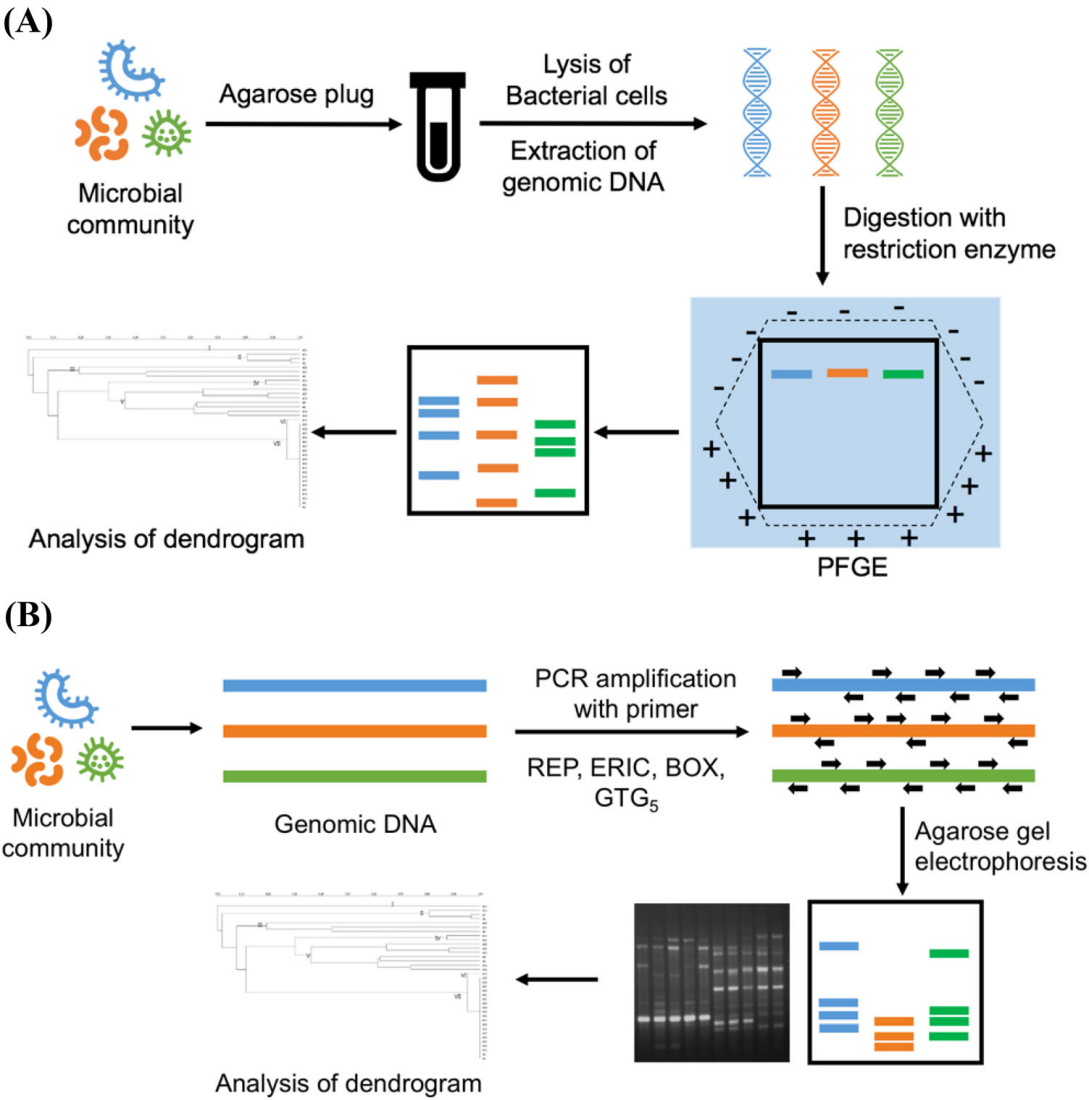
Schematic diagram for pulse field gel electrophoresis (**A**) and repetitive extragenic palindromic PCR (**B**)

Briefly, the bacterial DNA is immobilized into agarose blocks to avoid any mechanical damage to the DNA. Restriction endonuclease (rare cutter), which recognizes sequences of six-eight bps, digests the target DNA. Agarose blocks are placed into the migration gel wells after enzymatic digestion and subjected to an alternating pulse voltage gradient, resulting in a bacterial fingerprint (Fig. 3(A)). In PFGE, there is a periodic change in the electrical field orientation (Holzapfel et al., 2001). It is a gold standard method with good discriminatory power, reproducibility, and typeability (Chen et al., 2010). However, it is a labor-intensive and complicated procedure that demands skilled personnel for the analysis of the fingerprinting patterns (Neoh et al., 2019). In addition, this technique is prone to genetic instability, has minimal accessibility, and takes 3–4 d to complete (Wassenaar and Newell 2000).

PFGE has been efficaciously used for strain typing for the *Bifidobacterium* species, *Lb. plantarum, Lb. sakei*, *Lb. acidophilus*, *Lb. casei*, *Lb. delbrueckii*, *Lb. fermentum*, *Lb. helveticus*, and *Lb. rhamnosus* (Giraffa and Neviani 2000; Klein et al., 1998; Roussel et al., 1993; Roy et al., 1996; Sánchez et al., 2004). In a previous study, PFGE was found to be more discriminatory in identifying closely related *Lb. rhamnosus* and *Lb. casei* strains than ribotyping or RAPD (Tynkkynen et al., 1999).

## Repetitive extragenic palindromic PCR (Rep-PCR)

Rep-PCR has been widely applied for the typing of LAB strains. It works on the principle of producing highly specific genomic fingerprints by linking primers that correspond to interspersed repetitive specific DNA elements that are present at various locations within the LAB genome (Fig. 3(B)). PCR amplification of the distinct repetitive elements creates differently-sized DNA fragments that can be isolated by agarose gel electrophoresis, generating distinctive fingerprint patterns for different LAB strains (Fu and Li 2014). To determine genetic similarity, the fingerprint patterns are compared with each other. The different types of primers are Rep-PCR, enterobacterial repetitive intergenic consensus-PCR (ERIC-PCR), extragenic repeating PCR (BOX)-PCR, and (GTG)_5_-PCR sequences (Gevers et al., 2001). A combined approach, including (GTG)_5_-PCR fingerprinting and AFLP, has been established as a useful tool for LAB strain typing (Van Hoorde et al., 2008).

The advantages of Rep-PCR are that it has a short analysis time and high discriminatory power, requires a small amount of DNA, and is a procedure that is inexpensive and suitable for all LAB strains (Ranjbar et al., 2014; Singh et al., 2009). Moreover, its discriminatory power relies on the type of primer and number of repetitive sequences present in the LAB strain (Woo et al., 2006). Variability in PCR reagents and cycles and the conditions of gel electrophoresis can affect the reproducibility of Rep-PCR (Fu and Li 2014).

Our lab has applied this molecular tool for the characterization of the *Lb. brevis* (Kaur et al., 2018), *Leu. mesenteroides* (Kaur et al., 2017b), and *Leu. citreum* (Kaur et al., 2017c) strains obtained from different food products and locations in South Korea. Lee et al. used Rep-PCR to differentiate several *Lactobacillus* strains, namely *Lb. brevis*, *Lb. salivarius*, *Lb. reuteri*, *Lb. gallinarum*, and *Lb. panis,* at the strain level (Lee et al., 2012). Another study from Iran identified the probiotic strains in dairy products and Tarkhineh food using this approach (Tafvizi and Tajabadi Ebrahimi 2015). To identify bifidobacteria at the species, subspecies, and strain levels, BOXA1R primer has been proven to be a promising tool (Masco et al., 2003). Likewise, Jarocki et al. advocated that, in comparison to RAPD, ARDRA, and SDS-PAGE, BOX-PCR was found to be most effective in differentiating the *Bifidobacterium* strains at all taxonomic levels (Jarocki et al., 2016).

## Denaturing (D)/temperature (T) gradient gel electrophoresis (DGGE/TGGE)

**Fig. 4:**
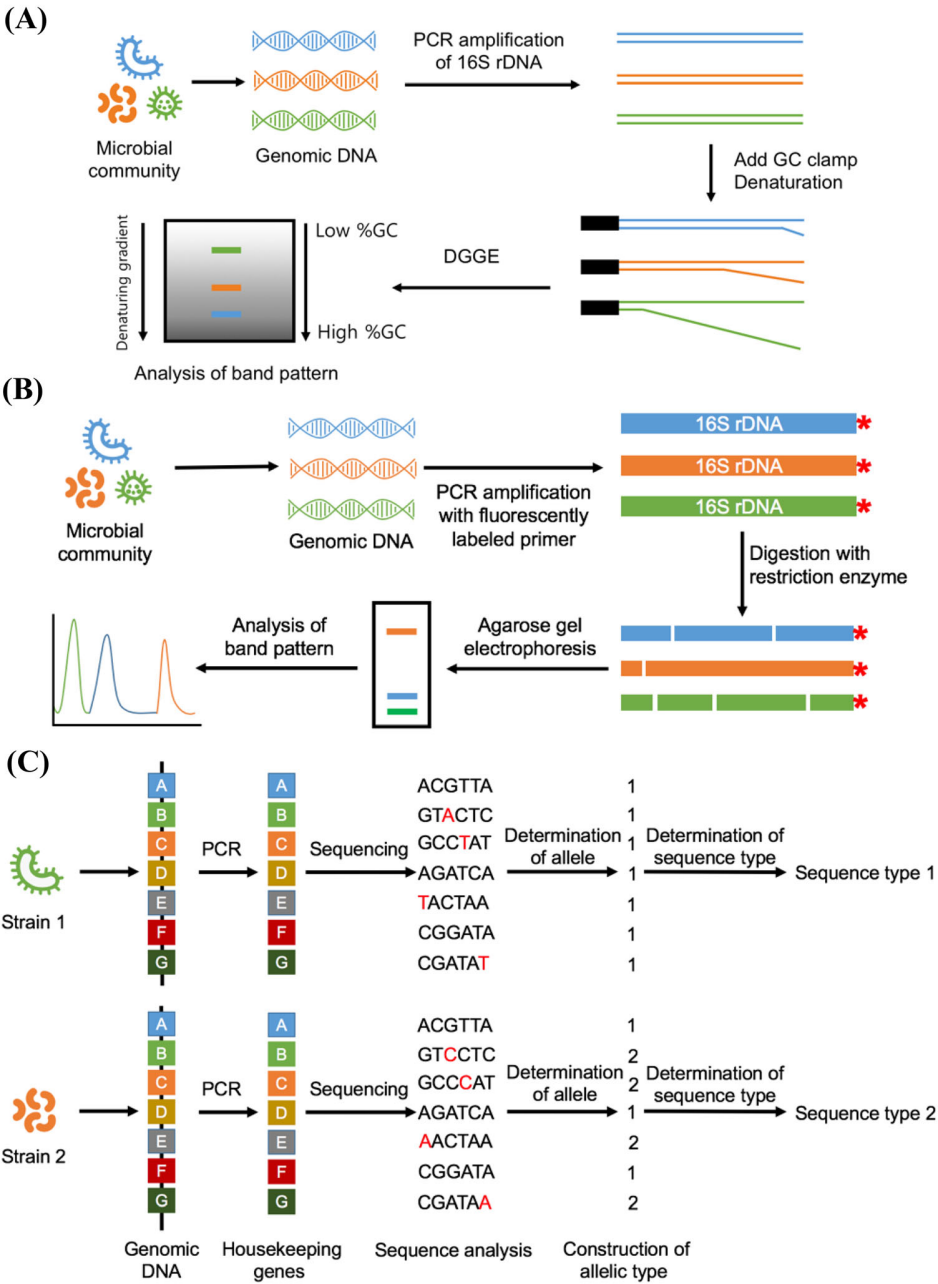
Schematic diagram for denaturing gradient gel electrophoresis (**A**), terminal (T)-restriction fragment length polymorphism (**B**), and multilocus sequence typing (**C**)

DGGE/TGGE is a culture-independent method, and primarily demonstrates the differences in DNA denaturation protocols. This tool has been used for the study and successive identification of specific bacterial species in a mixed bacterial population (Stefanis et al., 2016). The principle is based on the assumption that a denaturing or temperature gradient in polyacrylamide gel, which contains urea and formamide, can be used to separate 16S rDNA fragments of different sequences and similar or same lengths (Fig. 4(A)). Briefly, total DNA is extracted from a target bacterial population, and PCR is used to amplify the hypervariable regions within the 16S rDNA gene (Dimitrov 2019). One primer (GC clamp with a varying length of 30 to 50 bps and high GC content) is used to bind the denatured DNA fragments. The resulting PCR products are denatured based on their sequences since the GC-terminus is not denatured. The PCR products migrate to different locations in the gel owing to their unique melting patterns. The disparity in their DNA sequences is the reason for their varying melting profiles (Muyzer 1999). With proper selection of the analytical conditions and if the molecular weight of the PCR products is in the range of 200–600 bps, it is possible to differentiate PCR fragments even with a single nucleotide difference. Hence, the partly melted targets are separated due to the differences in their electrophoretic mobilities and visualized by staining. Ethidium bromide, SYBR Green I, and silver staining can be performed (Muyzer and Smalla 1998). For DGGE, the working temperature is maintained at 55 °C and 65 °C, while for TGGE, the temperature varies over the time required to develop the denaturing gradient.

Since no cultivation of bacteria is required, DGGE has a great advantage over a number of other methods. The disadvantages include poor detection of bacteria that are present in insignificant numbers in a community and incorrect estimation of the bacterial diversity due to the detection of the heteroduplex formed by the heterogeneous rRNA operons.

Hong et al. identified *Leu. mesenteroides*, *Leu. citreum*, *P. pentosaceus*, and *Leu. gelidum* as dominant bacteria in kimchi by microflora analysis (Hong et al., 2016). This tool was used to confirm the presence of probiotic strains in naturally fermented lactic acid products (Liang et al., 2018). In another study, *Lb. bulgaricus* was identified in intestinal samples (Dimitrov 2019) and *Lb. plantarum* was identified in red wine (based on *rpoB*) (Spano et al., 2007) using PCR-DGGE. This technique has also been applied to identify non-conventional LAB species, including *Lb. frumentii*, *Lb. acetotolerans*, *Lb. pontis* (recently reclassified as *Limosilactobacillus pontis*, Zheng et al., 2020), and *Lb. fructivorans* (recently reclassified as *Fructilactobacillus fructivorans*, Zheng et al., 2020) (Nishino et al., 2015; Wang et al., 2014; Wang and Nishino 2010; Wu and Nishino 2016). The identification of non-conventional LAB is required to elucidate their specific roles in various biological processes.

## Terminal (T)-restriction fragment length polymorphism (T-RFLP)

The conventional RFLP technique has low resolution, and therefore, small restriction fragments cannot be identified (Wang et al., 2010). As a substitute for the conventional RFLP technique, T-RFLP, founded on the basic steps of RFLP, can be used. This method includes extraction of DNA or RNA, PCR amplification, enzyme digestion, and fragment identification (Stefanis et al., 2016). The PCR amplification of a specific gene is usually performed with fluorescent-labeled primers [e.g. fluorescein amidite (6-FAM)], and it is followed by generation and separation of restriction fragments (usually four base cutter restriction enzymes are used) (Tabit 2016). The separated fluorescent-labeled terminal fragments of different sizes are determined using a DNA sequencer and analyzed by comparing either the bands or peaks of the T-RFLP runs to a database of known species (Fig. 4(B)). Similar to DGGE, T-RFLP is a nucleic acid-based tool that identifies specific microbial populations and develops a fingerprint of the microbial community. The strengths and shortcomings of this technique are identical to those of RFLP. This method is advantageous in providing a comparative bacterial profile of a sample of diverse bacteria. In addition, this technique does not require prior bacterial culturing for typing of a bacterial species from a mixed bacterial population (Tabit 2016). Additionally, profile accuracy can be increased by using more restriction enzymes.

In a previous study, the T-RFLP tool was used for identifying LAB at the species level during beverage fermentation (Bokulich and Mills 2012). Baniyah et al. used T-RFLP to assess the diversity of LAB in ileum broiler chicken. The study used restriction endonucleases *Msp*I and *Hae*III. Prebiotic bran-supplemented commercial feed, compared to *Rhizopus oryzae*-fermented bran, displayed a higher LAB diversity in the ileum of the animals. The predominant LAB was found to be the *Lactobacillus* sp. and *Lb. delbruecii* subsp. *bulgaricus* (Baniyah et al., 2018).

## Multilocus sequence typing (MLST)

MLST is a more effective tool for bacterial typing than the traditional 16S rRNA gene sequencing technique. MLST uses automated DNA sequencing, which involves the sequencing of internal fragments of the housekeeping gene loci (seven in number) of bacterial strains to characterize alleles (Maiden et al., 2013). The sequence of each fragment is compared with sequences (alleles) that were identified earlier at that locus, and allele numbers are assigned for the selected loci (Tanganurat et al., 2009). The combination of the seven genes governs the strain’s allelic profile, and each separate allelic profile is allocated a sequence type, which is used to categorize the target strain (Fig. 4(C)).

The MLST technique has been used to detect genetic differences in *Lb. plantarum* (de las Rivas et al., 2006; Xu et al., 2015), *St. thermophiles* (Delorme et al., 2017), the *Lb. casei* group (Bao et al., 2016; Diancourt et al., 2007; Feng et al., 2018), and *Oenococcusoeni* (De Las Rivas et al., 2004; Yu et al., 2018). Our research group used the MLST tool to differentiate the *Leu. mesenteroides*, *Leu. citreum*, and *Lb. brevis* strains (Sharma et al., 2017; Sharma et al., 2018a; Sharma et al., 2018b). MLST has not always been recorded advantageous in discriminating LAB strains. For example, in a previous study, it was found that PFGE showed higher resolving power than MLST in discriminating the *Lb. casei* strains. MLST differentiated 36 out of 40 *Lb. casei* strains, while PFGE differentiated all the 40 strains (Cai et al., 2007). Likewise, in a recent study, AFLP was found to be more efficient than MLST in differentiating the *Oenococcusoeni* strains (Yu et al., 2018).

MLST has a high discriminatory power, and it provides explicit results that can be compared to those obtained in different laboratories. Nevertheless, it has practical disadvantages, including high cost and restricted accessibility (Sullivan et al., 2006). Furthermore, since housekeeping genes are conserved in nature and highly variable in different bacterial species, MLST may not adequately discriminate unrelated isolates.

## Real-time PCR

**Fig. 5:**
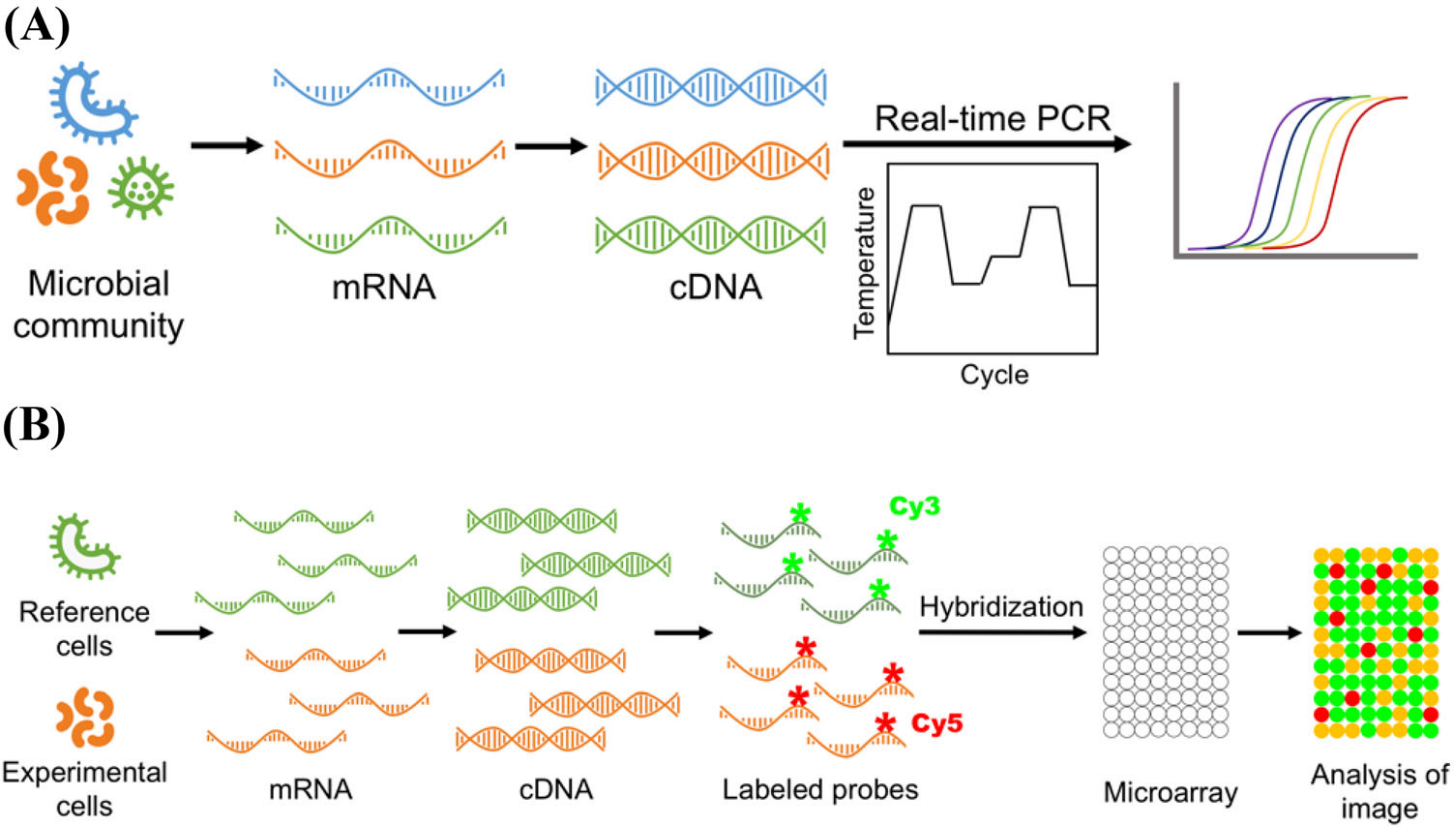
Schematic diagram for real-time PCR (**A**) and microarrays (**B**)

The most important breakthrough in the application of PCR was observing DNA amplification based on the fluorescence emission in real-time (Holland et al., 1991). Real-time PCR can be used based on the goal of the study: absolute (standard curve) or relative (comparative threshold method) quantification(Arya et al., 2005). Absolute quantification depends on the preparation of a standard curve using diluted template samples of known concentrations, including a plasmid containing the cloned gene of interest, a synthetic oligonucleotide (single sense), genomic DNA, cDNA, total RNA, or in vitro transcripts (Arya et al., 2005). The standard curve approach is used to quantify viral load or the exact level of the DNA template in the samples (Kralik and Ricchi 2017). Relative quantification is based on mathematical calculations that are used to quantify the relative expression of a target compared to that of a control (Arya et al., 2005). It is used to analyze the gene expression and comparative amount of DNA (Fig. 5(A)).

Real-time PCR is a DNA-based technique that has been widely used to quantify LAB species from various samples, including food, milk, and feces. It is based on the principle of measuring the intensity of the fluorescence of the product generated during each amplification cycle of the PCR process, and this intensity is directly proportional to the increase in the amount of the amplicon at that specific time (Kralik and Ricchi 2017). Fluorescent-labeled oligonucleotide probes [hydrolysis probes (TaqMan^®^ assay), Molecular Beacons and Scorpions] and non-sequence-specific double standard DNA fluorescent intercalating dyes (SYBR Green I) (Mackay et al., 2002) are the two most widely used methods to monitor the real-time target sequence amplification. Several different PCR instruments and probe formats are available for real-time detection. The probe-based approach shows promise owing to the lesser chance of developing non-specific PCR products (primer-dimers) and higher specificity facilitated by the extra oligonucleotide (Hein et al., 2001; Kubista et al., 2006). However, SYBR Green I dye can be used to identify a target from a mixed population using PCR. In addition, real-time PCR enables precise quantification of templates of more than 10^7^-fold dynamic range (Stefanis et al., 2016).

The principle benefit of this method is that it is sensitive and suitable to quantify LAB. It is a superior tool compared to conventional PCR-based techniques because it circumvents the necessity for post-PCR handling. In addition, no tiresome selective bacterial culturing is required, as demanded by other genotypic methods (Sattler et al., 2014). Furthermore, the performance of real-time PCR can be significantly improved in a multiplex setup, in which more sequences are simultaneously targeted (Bottari et al., 2013). This can be conducted using the new closed-tube structures that decrease the possibility of contamination during real-time PCR. The biggest disadvantage, however, is the failure to differentiate dead bacteria from the living. This can occur because DNA can be amplified from dead cells too (Josephson et al., 1993; Justé et al., 2008; Kralik and Ricchi 2017). Another limitation is that in the reaction tube, the target DNA amplicons are restricted due to the different natures of the illuminating light sources and fewer fluorescent dyes (Mackay et al., 2002). A modification of this method, multiplex real-time PCR, permits the amplification of more than one target in a single reaction using distinct reporters with different fluorescent spectra.

A previous study used real-time PCR to enumerate *Lb. lactis* subsp. *cremoris*, *Lb. lactis* subsp. *lactis,* and the *Leu.* sp. relatively for the first time, and these results were compared to those obtained by flow cytometry-fluorescence in situ hybridization (FLOW-FISH). On comparison, we found that quantitative PCR was more flexible than FLOW-FISH (Friedrich and Lenke 2006). Bottari et al. documented a multiplex real-time PCR system used to directly identify the thermophilic LAB, including *Lb. delbrueckii*, *Lb. helveticus*, *St. thermophilus*, and *Lb. fermentum* in undefined starter cultures of hard cooked cheeses (Bottari et al., 2013). This tool was found to be more effective and faster than the length-heterogeneity PCR and FISH methods (Bottari et al., 2013). Furet et al. used real-time PCR for the specific detection and quantification of *Lb. acidophilus*, *Lb. delbrueckii*, *St. thermophilus*, *Lb. johnsonii*, *Lb. rhamnosus*, and *Lb. paracasei* in commercial fermented milk products (Furet et al., 2004). Recently, real-time PCR has been used for the quantification of thermophilic *Lb. delbrueckii* subsp. *bulgaricus* and *St. thermophilus*, which are commonly used for milk fermentation and cheese ripening (Stachelska and Foligni 2018). Another study described the use of strain-specific real-time PCR for enumerating the *Lb. reuteri* strains in a chicken feed (from three separate locations) in the three sections (ileum, jejunum, and caecum) of the gastrointestinal tract of birds of different ages (Sattler et al., 2014). Pontonio et al. used real-time PCR for the differentiation of LAB in bread that was made with or without sourdough (Pontonio et al., 2017). In a recent study in China, real-time PCR was utilized to assess the LAB biodiversity in 86 fermented milk products. Among the 705 LAB species that were identified, *Lb. delbrueckii* subsp. *bulgaricus*, *Lb. helveticus,* and *Lb. fermentum* were the major species. Significantly different microbiota were found in cow and yak milk samples (Mo et al., 2019).

## Microarrays

Microarray is a novel genomic tool widely used to investigate transcriptional profiles across genomes (Rick et al., 2001). Generally, the types of microarrays include cDNA microarrays, oligonucleotide microarrays, and serial analysis of gene expression. Microarrays work on the principle that complementary sequences bind to each other. Briefly, a DNA microarray experiment consists of array fabrication, probe preparation, hybridization, and data analysis. The labeled [usually with red (Cy5) and green (Cy3) dyes] cDNA interacts with a particular immobilized probe. The signal generated from this interaction provides information on the type of RNA present in the undetermined target specimen (Fig. 5(B)). In simple terms, microarray provides diagnostic data, which is information on the gene expression. Hence, microarray can be applied to analyze the gene expression profiles across genomes and similarity or dissimilarity of the genome levels of diverse microorganisms. Microarrays can be differentiated based on their features, including surface support, probe type, and approach to recognize the target (Miller 2011).

The genomic data have been used to design numerous species-specific microarrays that permit the monitoring of the expression of all genes of a single LAB species and observation of the remarkable metabolic functions during monoculture experiments (Kuipers et al., 2002; Pieterse et al., 2005). Furthermore, Weckx et al. identified and validated a species-independent microarray of 406 functional genes that targeted LAB to evaluate the gene expression levels. The study demonstrated the use of gene-specific oligonucleotide design algorithm to hybridize multiple species of LAB (Weckx et al., 2009). Another study described the anti-colitic effect of a mixture of LAB strains, including *Bif. longum*, *Lb. brevis*, and *Lb. suntoryeus,* in a colitic ICR mouse (induced by dextran sulfate) using cDNA microarray and qPCR. The results showed that the supplemented mixture ameliorated colitis by reducing the expression levels of tumor necrosis factor (TNF)-α, interleukin (IL)-1β, and cyclo-oxygenase (COX)-2 (Lee et al., 2009). The reformed functional potential of a mutated *Lac. lactis* strain was analyzed based on its genetic and microarray information (Chen et al., 2015). While microarrays are still considered important and cost-effective techniques for the analysis of different microbial communities, the next-generation sequencing (NGS) tools are supplanting them with each passing year (Ledford 2008).

## Mass spectrometry (MS)

**Fig. 6:**
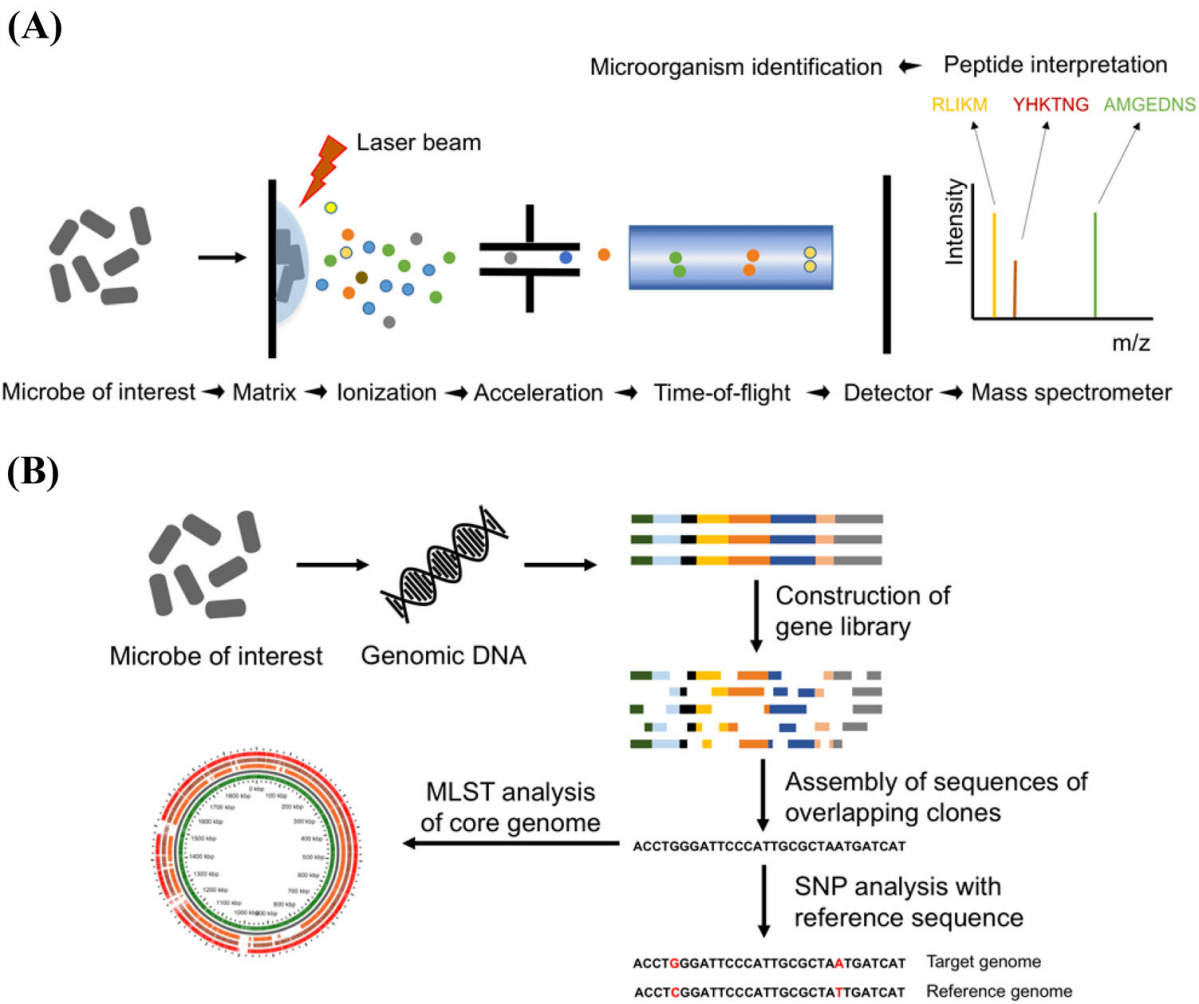
Schematic diagram for mass spectrometry (**A**) and whole-genome sequencing (**B**)

For bacterial typing, there are three basic platforms of MS, including matrix-assisted laser desorption/ionization-time of flight (MALDI-TOF) MS, liquid chromatography (LC)-MS/MS, and targeted LC–MS/MS (Cheng et al., 2016; Ferranti 2004; Vinusha et al., 2018). In MALDI-TOF, the target bacteria are treated with laser radiations, which are transferred through the matrix. Different ions that are generated move towards a detector at varying speeds. The obtained fingerprints are compared for the identification and characterization of bacterial strains. The use of MALDI-TOF MS is advantageous in the analysis of proteins of large sizes. The LC–MS/MS approach to fingerprinting is based on peptide analysis. For peptide generation, proteins from the target bacteria are concentrated, converted into peptides using enzymes (e.g., trypsin), separated using LC, ionized using electrospray ionization (ESI), and analyzed using MS. The ionized peptides are additionally fragmented, and the obtained sequences are used for identifying the bacteria (Vinusha et al., 2018) (Fig. 6(A)). Targeted LC–MS/MS uses standards labeled with stable isotopes for the identification and quantification of the target peptide. The applications of the MS tool include obtaining information on the peptide mass, new amino acid sequence data, and information on post-translational modifications in proteins (Table 1).

**Table 1:** Molecular typing tools and their important characteristics

Molecular tool	Reproducibility	Discriminatory power	Applicability	Interpretation	Duration (day)	Stability	Repeatability	Cost	Ease of use
RT	High	Low	Y*	Moderate	1 to 4	Good	High	High	Labor-intensive
ARDRA	High	Poor to moderate	Y	Easy	1–2	Good	High	Low to moderate	Simple
RAPD	Moderate	Good	Y	Easy	1	Moderate	Moderate	Low	Simple
AFLP	Good to excellent	High	Y	Moderate	2	Good	High	moderate to high	Poor to moderate
PFGE	High	High	Y	Moderate	3	Good	High	moderate	Labor-intensive
Rep-PCR	Good to excellent	Moderate to excellent	Y	Moderate to easy	1	Good	Good to excellent	Low to moderate	Good
DGGE/TGGE	Moderate	High	Y	Moderate	> 3	Good	Good to excellent	Moderate	Moderate
T-RFLP	High	Poor to moderate	Y	Easy	1–2	Good	High	Low to moderate	Simple
MLST	High	Moderate to good**	Y	Moderate	> 3	Good	High	High	Difficult
Real-time	High	High	Y	Moderate	1	Good	High	High	Moderate
Microarray	High	Good to excellent	Y	Moderate	1 to 3	Moderate	High	High	Moderate
MS	High	Low to moderate	Y	Difficult	> 3	Good	Good to excellent	High	Difficult
WGS	High	High	Y	Difficult	< 2***	High	High	High	Difficult

Y, yes; RT, ribotyping; ARDRA, amplified ribosomal DNA restriction analysis; RAPD, random amplified polymorphic polymorphic DNA; AFLP, amplified fragment length polymorphism; PFGE, pulse-field gel electrophoresis; Rep-PCR, repetitive extragenic palindromic-PCR; DGGE/TGGE, denaturing/temperature gradient gel electrophoresis; T-RFLP, terminal (T)-restriction fragment length polymorphism; MLST, multilocus sequence typing; Real-time; Real-time PCR; MS, mass spectrometry; WGS, whole genome sequencing (Adapted and modified from Ferrari et al., 2017; Mughini-Gras et al., 2019; Ranjbar et al., 2014)

*Universal applicability

**Based on the type and number of housekeeping genes

***Depending upon the number of strains and type of sequencers

The advantages of the MS technology are that it is consistent, robust, and cheap, requires only a small amount of the sample, consumes less time, and manages data better. The disadvantages include database dependency, requirement of trained staff, and inability in identifying non-viable microbes.

The technique MALDI-TOF MS has been applied to identify LAB from French Maroilles cheese. MALDI-TOF MS identified 105 and 92 LAB strains from Maroilles cheese synthesized using raw milk and pasteurized milk, respectively. Among the three identified genera, namely *Lactobacillus*, *Leuconostoc*, and *Enterococcus*, *Lactobacillus* was the most prominent genus with the *Lb. brevis*, *Lb. plantarum*, *Lb. curvatus* (recently reclassified as *Latilactobacillus curvatus*, Zheng et al., 2020), *Lb. paracasei*, *Lb. rhamnosus*, *Lb. parabuchneri*, and *Lb. fructivorans* species (Nacef et al., 2017). Likewise, this tool was applied to identify LAB species from two fishes, namely *Oreochromis niloticus* and *Mugil cephalis*. The identified genera included *Leuconostoc*, *Enterococcus*, *Lactococcus*, and *Vagococcus* (El-Jeni et al., 2019). Another recent study used MALDI-TOF MS to identify nonstarter LAB isolates from four artisanal kinds of cheeses, namely Kefalotyri, Anthotyro, Xynotyri, and Touloumotyri, synthesized from raw goat and sheep milk. The identified LAB species included *Lb. brevis*, *Lb. rhamnosus*, *Lb. plantarum*, *Lb. paracasei*, *Leu. mesenteroides*, *Enterococcus faecium*, *Lac. lactis*, and *P. pentosaceus* (Gantzias et al., 2020). Albesharat et al. assessed the LAB strains in breast milk, feces of mothers and babies, and local foods (fermented) using genotypic tools such as MALDI-TOF MS, 16S RNA gene sequencing, and RAPD (Albesharat et al., 2011). They identified the *Lactobacillus*, *Streptococcus*, *Pediococcus*, *Enterococcus*, and *Weissella* genera. Specifically, *Lb. brevis, Lb. fermentum*, *Lb. plantarum*, and *P. pentosaceus* were identified in all the sources (Albesharat et al., 2011).

In a recent study, MALDI-TOF MS and 16S rRNA gene sequencing were used to identify LAB species from by-products of fruits (mango, strawberry, Barbados cherry, and soursop). The identification match for both techniques was 86%. The predominant genus was *Lactobacillus*, which included the *Lb. brevis*, *Lb. fermentum*, *Lb. pentosus*, *Lb. paracasei*, *Lb. plantarum*, *Lb. casei*, and *Lb. nagelii* (recently reclassified as *Liquorilactobacillus nagelii*, Zheng et al., 2020) (Garcia et al., 2016). Russo et al. applied the nano-LC–ESI–MS/MS tool to identify LAB in whey starter cultures by utilizing species-specific peptide markers at the genus, species, and subspecies levels for the first time (Russo et al., 2019).

## Whole-genome sequencing (WGS)

The introduction and availability of NGS and 3rd generation technology, including WGS and high-throughput sequencing, has resulted in an unprecedented rise in the volume of sequencing-based data (Kwong et al., 2015). The sequencing of the complete genome sequences of diverse LAB strains enables introduction of new approaches that can be used to deduce the evolutionary and divergence relationships among the strains (Coenye and Vandamme 2003). WGS is a powerful approach used to characterize strains accurately and interpret functions of LAB at the genome level (Buron-Moles et al., 2019). Furthermore, WGS may provide information on the antimicrobial resistance of the LAB strains. The first LAB genome was released in 2001 (Bolotin et al., 2001). Subsequently, full genome sequences of many other LAB species have been made readily accessible (Chenoll et al., 2015; Inglin et al., 2018; Sun et al., 2015; Wu et al., 2017; Zheng et al., 2015).

WGS technology is gradually substituting customary microbial typing and characterization methods and offering faster and more accurate answers (Jagadeesan et al., 2019). WGS is gaining attention as a potential approach for the surveillance of food-borne illnesses (Allard et al., 2016; Ashton et al., 2016). Currently, there are two methods that can be used to analyze WGS data to determine the similarities among the strains: single nucleotide polymorphism (SNP)- and gene by gene-based approaches. Investigation of WGS data through either method is a dynamic process, which involves several steps to generate outcomes, such as allele matrices and phylogenetic relationships (Fig. 6(B)).

In the SNP-based method, the target genome is compared with the reference genome to obtain information on the nucleotide differences. On the other hand, the gene by gene-based method, an alternate approach to traditional MLST, is classified into whole genome (wg) MLST and core genome (cg) MLST, and can be used to analyze the genetic relatedness among the LAB strains (Schürch et al., 2018). In this approach, compared to the conventional MLST method, alleles are assigned using big data pools that are generated through multiple comparisons between genomes or several genes and the curated genomic information to corroborate reproducibility among the results obtained by different laboratories. A recent cg study assessed 56 LAB genomes to trace the genetic determinants of the carbohydrate metabolism in the strains. The study found that 219 single-copy genes were shared among these 56 strains (Buron-Moles et al., 2019). Kant et al. reported 383 core gene sets by comparing 20 *Lactobacillus* genomes (Kant et al., 2011). The genome data offered a greater resolution than that obtained using the other previously described genotyping tools. However, there are some blockades to the extensive employment of WGS in different laboratories. This method requires expensive equipment and a bioinformatician to handle the technical data. Furthermore, while many WGS analyses can be theoretically conducted on standard computers, the computational power required for processing and analyzing large numbers of genomes is important (Kwong et al., 2015).

Probiotics have developed into a significant health concern worldwide. As previously described, several typing tools have been established to identify and classify probiotics or LAB strains and find a genetic link among these beneficial microbes. The conventional phenotypic approaches have merits and pitfalls that affect their utility. Though the use of a wide range of molecular techniques has facilitated the typing of LAB strains, a polyphasic approach that consists of two or more typing tools is ideal for the correct documentation of LAB strains. Furthermore, development and access to the NGS techniques, including WGS, has allowed for the analysis of the differences in DNA sequences. It can be anticipated that in the near future, these technologies will replace traditional typing tools. However, these sequencing-based techniques are available only in well-equipped laboratories and require highly qualified personnel. In our opinion, for the analysis of a limited number of strains, less innovative methods will still be used, and hence, the development of easy, quick, and cost-effective techniques with high discriminatory power is necessary.

## Abbreviations

AFLP: Amplified fragment length polymorphism
ARDRA: Amplified ribosomal DNA restriction analysis
*Bif*.: *Bifidobacterium*
DGGE: Denaturing gradient gel electrophoresis
ERIC-PCR: Enterobacterial repetitive intergenic consensus-PCR
ESI: Electrospray ionization
FLOW-FISH: Flow cytometry-fluorescence in situ hybridization
*Lac.*: *Lactococcus*
*Lb*.: *Lactobacillus*
*Leu*.: *Leuconostoc*
MALDI-TOF: Matrix-assisted laser desorption/ionization-time of flight
MLST: Multilocus sequence typing
MS: Mass spectrometry
NGS: Next-generation sequencing
*P.*: *Pediococcus*
PFGE: Pulse field gel electrophoresis
RAPD: Random amplified polymorphic DNA
RFLP: Restriction fragment length polymorphism
Rep-PCR: Repetitive extragenic palindromic PCR
*S*.: *Saccharomyces*
SNP: Single nucleotide polymorphism
*St*.: *Streptococcus*
TGGE: Temperature gradient gel electrophoresis
WGS: Whole-genome sequencing
